# Association between cadmium exposure and renal cancer risk: a meta-analysis of observational studies

**DOI:** 10.1038/srep17976

**Published:** 2015-12-11

**Authors:** Ju kun Song, Hong Luo, Xin hai Yin, Guang lei Huang, Si yang Luo, Du ren Lin, Dong Bo Yuan, Wei Zhang, Jian guo Zhu

**Affiliations:** 1 Department of Oral and Maxillary Surgery, Gui Zhou provincial people’s hospital, Guiyang, China; 2 Department of Urology, Gui Zhou provincial people’s hospital, Guiyang, China

## Abstract

Cadmium (Cd) is a widespread environmental pollutant and has been a recognized carcinogen for several decades. Many observational studies reported Cd exposure might be one cause of renal cancer. However, these findings are inconsistent. We conducted a meta-analysis to evaluate the relationship between cadmium exposure and renal cancer risk. A comprehensive PubMed and Embase search was conducted to retrieve observational studies meeting our meta-analysis criteria. A combined odds ratio (OR) and corresponding 95% confidence interval (CI) were applied to assess the association between Cd exposure and renal cancer risk. The meta-analysis showed that a high Cd exposure significantly increased renal cancer 1.47 times (OR = 1.47; 95% CI = 1.27 to 1.71, for highest versus lowest category of cadmium categories). The significant association remained consistent when stratified by geographic region and gender, however mixed results were produced when stratified by sample size, study design, NOS score, adjustment for covariates, effects measure, and exposure type. Our results indicated that a high Cd exposure was associated with increased renal cancer risk and the association was higher for occupational exposure compared with non-occupational exposure. This meta-analysis suggests that a high Cd exposure may be a risk factor for renal cancer in occupational population.

In 2015, a total of 61,560 new cases of renal cancer were diagnosed with 14,080 deaths in the USA alone, making renal cancer the sixth leading cause of cancer deaths^1^. The prevalence of renal cancer has been increasing by approximately 2–4% per year for the last two decades worldwide^2^. The advancement in imaging diagnoses and early screening do not fully explain this trend^3^. Among the African-American population, the incidence of renal cancers has shown a more rapid increase. Furthermore, studies estimate renal cancer will be a major concern in the male population due to a recent rise in documented cases. During the past two decades, established renal cancer risk factors, including tobacco smoking, heavy alcohol drinking, hypertension, obesity, and use of phenacetin-containing drugs were well documented as predominant etiologic factors for renal cancer^3^.

Cadmium (Cd) is a toxic heavy metal harmful to human health found naturally at low levels in rocks and soil. Cd is accumulated in the kidney cortex and is one cause of end-stage renal disease^4^,^5^. Recently, numerous observational studies were conducted to evaluate Cd exposure effects on renal cancer susceptibility, which showed positive^5^,^6^,^7^,^8^,^9^,^10^ and null associations^11^,^12^,^13^. However, these studies had small sample sizes, which might prevent any capacity to detect an effect. Therefore, given the increased diagnosis of Cd exposure and poor prognosis of renal cancer, risk factors for renal cancer development would have a substantial impact on public health. Therefore, the objective of our study was to assess any association between Cd exposure and renal cancer risk by conducting cohort, case control, or cross-sectional meta-analysis. In addition, clarifying a relationship might emphasize the importance of considering additional preventative methods for renal cancer. The study was reported following the Preferred Reporting Items for Systematic Reviews^14^.

## Results

### Literature search, study characteristics, and quality

**Figure 1:**
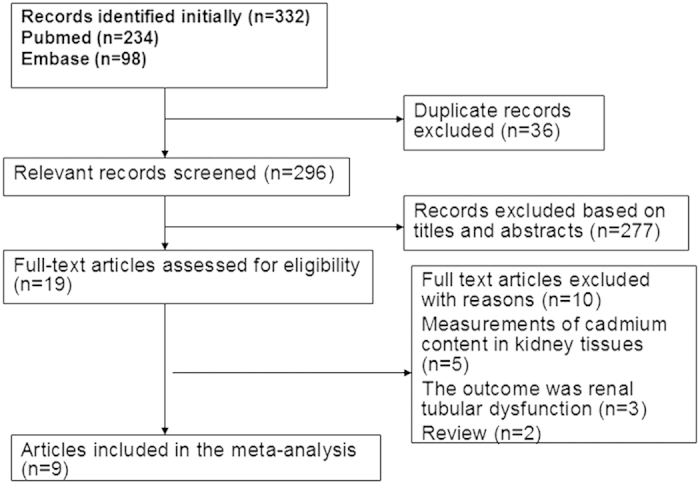
Flow diagram of the literature included.

Following development of our search strategy, 332 records were initially identified. Thirty-six duplicate studies were excluded; 296 were subsequently screened, and 139 were excluded because titles and abstracts indicated the studies did not fit our criteria. Nineteen full-text articles were reviewed for further assessment. Five articles were excluded because Cd content in kidney tissues was measured^15^,^16^,^17^,^18^,^19^, three were not appropriate due to a renal tubular dysfunction outcome^20^,^21^,^22^, and two were review studies^23^,^24^. Finally, nine articles met the meta-analysis criteria and were included (Fig. 1).

The descriptive data for all articles included in the study are summarized in Table 1. One article reported two different ethical approaches, hence it was considered two individual studies^10^. Therefore, nine total articles, ten case-control studies (6,013 incident cases and 21,104 controls) and one cohort study (9 renal cancer cases and 1,732 participants) contributed to the meta-analysis. The number of renal cancer patients ranged from 9 to 1,723 across all included studies. The cases were histologically, pathologically, or clinically confirmed as renal cancer. Four studies were based in Europe^6^,^7^,^10^,^12^, four in North American^5^,^8^,^11^,^13^, and one in a mixed population(Austrlia, Denmark, Germany, Sweden, and United States)^9^. The articles were published from 1976 to 2014. Seven studies were designed to evaluate renal cancer odds ratio (OR)^5^,^6^,^7^,^8^,^10^,^12^, two evaluated relative risk (RR)^9^,^13^, and one evaluated hazard risk (HR)^11^. All studies investigated women and men, with the exception of one study, which reported results for the association of renal cancer in men^5^. Seven studies adjusted a group of variables for conventional risk factors in renal cancer, including age, gender, geographic area, and smoking^8^,^9^,^10^,^11^,^12^,^13^, whereas the other studies did not control for other confounding factors^5^,^6^,^7^. Eight studies reported an association between occupational Cd exposure and renal cancer risk^5^,^6^,^7^,^8^,^9^,^10^,^12^,^13^, while a subject in one study was related to a non-occupational population^11^.

The Newcastle-Ottawa Scale (NOS) was used to assess the quality of studies included in the meta-analysis (Table 2). The median NOS score was 5.7 (range: 4 to 7).

### Overall analysis

**Figure 2:**
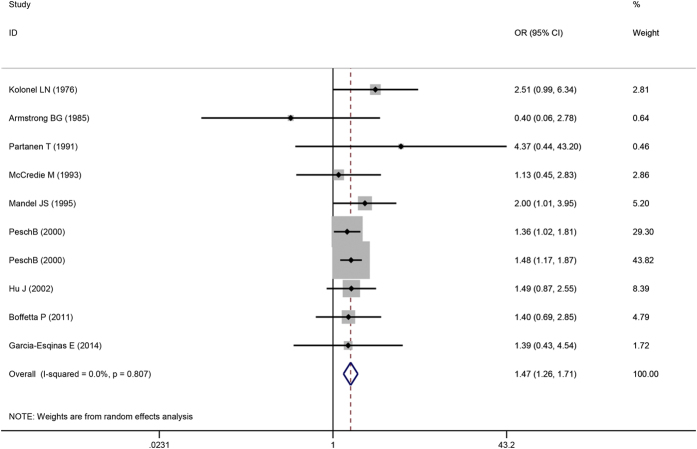
Forest plot for the association between cadmium exposure and renal cancer risk.

Overall OR estimates for each study were combined to determine total risk estimates using a fixed-effects model (OR = 1.47; 95% CI = 1.27 to 1.71, *P *= 0.000) with low heterogeneity (*P*_*heterogeneity*_ = 0.807, *I*^2^ = 0%). Two estimates from the same study were shown; the study provided separate analyses for two case-control studies, which were depicted separately in the figure (Fig. 2).

### Subgroup and sensitivity analysis

**Figure 3:**
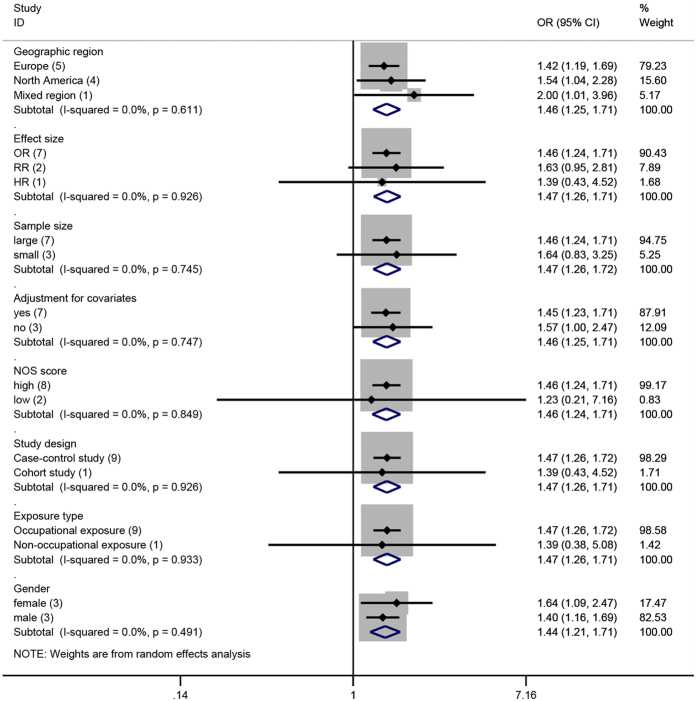
Subgroup analysis for cadmium exposure and renal cancer risk.

We performed sensitivity analysis to test the stability and robustness of the association, where one study at a time was omitted and the combined OR was computed for the remaining studies. Exclusion of any single study did not notably effect the overall combined OR, which ranged from 1.44 (95% CI, 1.23–1.69) to 1.51 (95% CI, 1.26–1.82); in addition, low heterogeneity was observed. Subgroup analysis was also performed (Fig. 3, Table 3). A statistically significant association between Cd exposure and renal cancer risk was not altered by geographic region and gender; however, mixed results were observed when stratified by sample size, study design, NOS score, adjustment for covariates, effect size, and exposure type. A significant association was found in case-control studies (OR = 1.47, 95% CI: 1.26–1.72), but not in cohort studies (OR = 1.39, 95% CI: 0.43–4.45). When stratified by exposure type, the association was significant between occupational exposure populations (OR = 1.47, 95% CI: 1.26–1.72), but not among non-occupational exposure populations (OR = 1.39, 95% CI: 0.43–4.45). Compared with a low NOS score (OR = 1.23, 95% CI: 0.21–7.11), the association was higher among studies with a high NOS score (OR = 1.46, 95% CI: 1.24–1.71). When stratified by different effects measures, the association was significant among OR studies, but RR or HR risk estimate studies showed a lack of significance. When stratified by sample size, a significant association was detected among studies with patient samples of ≥100 cases (OR = 1.46, 95% CI: 1.24–1.71), but a significant association was not observed for patient samples of <100 cases (OR = 1.64, 95% CI: 0.83–3.25).

### Publication bias

**Figure 4:**
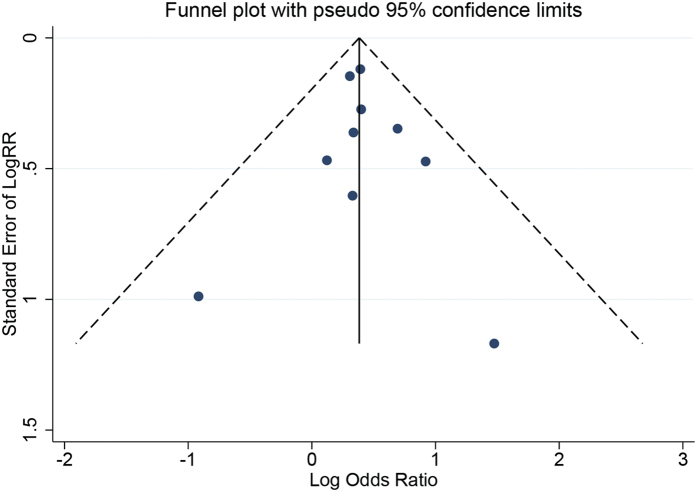
Funnel plot for publication bias analysis results.

Evidence of publication bias was not detected using Egger’s test (*P *= 0.759) and funnel plot symmetry was observed in the meta-analysis (Fig. 4). The results remained unaltered after the trim and fill analysis (OR_fixed_ = 1.47, 95% CI: 1.26–1.71; OR_random_ = 1.47, 95% CI: 1.26–1.71), suggesting stable results.)

## Discussion

Cadmium was widely used in industry until one decade ago, when its health risks were recognized, however it is distributed naturally at low levels throughout the environment. Recently, increasing evidence established a link between cadmium exposure and prostate cancer^25^,^26^, breast cancer^27^,^28^,^29^, pancreatic cancer^30^,^31^, and lung cancer^32^,^33^. In addition, many current observational studies reported positive associations between exposure to Cd and renal cancer risk. However, these studies had a modest sample size and the association magnitude was variable among studies, with OR ranging from 0.4 (95% CI: 0.05–2.41) to 4.37 (95% CI: 0.44–43.00) and the confidence interval was notably wide. Therefore, the magnitude was limited due to the low precision in risk estimates. These epidemiological studies showed the absence of a comprehensive assessment in cadmium exposure. Therefore, we conducted a comprehensive retrospective meta-analysis to investigate any association between cadmium exposure and renal cancer risk.

To the best of our knowledge, this is the first meta-analysis to explore the role of cadmium exposure in renal cancer patients. The overall results of the present meta-analysis of ten observational studies using a fixed-effects model provided evidence that a high Cd exposure was associated with increased renal cancer risk. The pooled estimates were robust across the sensitivity and subgroup analyses, and publication bias was not detected. The conclusions from combined estimates were more reliable than from a single study because the overall OR was based on a large sample size and exhibited sufficient power.

Several mechanisms are responsible for the carcinogenesis of Cd exposure. Recently, several observational studies using *in vitro* cell culture and *in vivo* animal studies demonstrated a proliferative and carcinogenic effect of Cd on various cancers^34^,^35^,^36^. A major portion of Cd is bound to metallothionein proteins. These proteins, which have low molecular weight, play a vital role in essential-metal homeostasis^37^. The cadmium-metallothionein compound is disseminated in different organs and subsequently reabsorbed in kidney tubules. A Cd excretion mechanism is not present in the human body, resulting in Cd accumulation. Cadmium half-life in the human kidney cortex is ∼10–30 years^38^. Proto-oncogene activation, tumor suppressor gene inactivation, cell adhesion disruption, and DNA mismatch repair inhibition are some cellular and molecular mechanisms indicated in cadmium carcinogenicity^39^,^40^,^41^. These processes are involved in cancer development.

The present meta-analysis exhibited several strengths. First, the meta-analysis was the first to investigate an association between Cd exposure and renal cancer risk. Second, the large sample size improved the risk estimate accuracy and resulted in well-founded conclusions based on the meta-analysis. Third, the analysis employed multivariable-adjusted risk estimates to minimize the confounding factors that influenced Cd exposure levels. The studies with adjusted risk estimates accurately reflected the association between Cd exposure level and renal cancer risk. Fourth, low heterogeneity was detected across the studies and publication bias was not observed.

Nevertheless, some limitations should be considered in the present meta-analysis. First, although a case-control study is the most appropriate design for toxicity exposure (e.g., occupational or environmental) causing rare health events, this design has inherent limitations, such as selective and recall or memory bias. Second, confounding factors, including co-exposure to other toxic chemicals and lifestyle factors (e.g., lead, asbestos, arsenic, tobacco and/or alcohol consumption) are difficult to control in a meta-analysis. Third, the small number of studies included in a meta-analysis limits the ability to draw robust conclusions, particularly in the subgroup analysis. Finally, the included studies were only distributed in Europe and North America. Therefore, further study should investigate the association between Cd exposure and renal cancer susceptibility among Caucasian, African, and Asian populations or additional ethnicities on other continents.

The following factors should be considered for further studies. First, in the meta-analysis, we found only one study validated Cd exposure levels. It is vital to estimate Cd exposure levels in urine and blood, proportional to the body’s tolerance, which reflect long-term Cd exposure levels. The precision of observational Cd related renal cancer hypothesis studies could be greatly improved by incorporating Cd exposure biomarkers. Therefore, future studies should examine Cd levels in urine and blood as a method to assess long-term Cd exposure. Second, most studies we examined investigated the association between Cd occupational exposure and renal cancer risk. Therefore, the results of our meta-analysis should only be used to infer Cd under occupational conditions leading to increased renal cancer risk. Compared with the general population, Cd levels are typically higher in certain industries as a component of an occupational population where Cd is present (e.g., nickel batteries, pigments, and soldering alloys). However, Cd exposure levels in the general population are usually low. Therefore, further studies are needed to confirm the association among the non-occupational (Cd-exposed) population. Finally, studies we analyzed in the meta-analysis did not examine whether an association between Cd exposure and renal cancer risk differed among anatomical or histological sub-sites within the body.

In summary, the meta-analysis suggests that a high cadium exposure may be a risk factor for renal cancer in occupational population. Further study should be conducted to determine whether a low level Cd exposure in general population was associated with increased risk of renal cancer.

## Methods

### Data source and search strategy

A comprehensive search was performed using PubMed and EMBASE databases to retrieve all potentially related studies up to June 2015. We employed the following search strategies (i.e., search terms) without limitations: “renal cancer” or “kidney cancer” or “renal cell cancer” or “renal cell carcinoma” combined with “cadmium”. The search was limited to human subjects. The previous review and related article references were manually screened to identify other potentially eligible studies.

### Eligibility criteria and study selection

Studies were considered eligible for inclusion in the meta-analysis if they met the following criteria: (1) Cd was the heavy metal of human exposure; (2) the outcome was renal cancer risk; (3) the study design was cohort, case control, or cross-sectional; and (4) the relative risk (RR), odds ratio (OR), or hazard risk (HR) with corresponding 95% confidence interval (CI) were reported or provided sufficient data to estimate crude OR, RR, or HR with corresponding 95% CI. If the included population was duplicated in more than one study, only data from the study with the most comprehensive information was included.

### Data extraction and quality assessment

Two authors (JKS and XHY) independently extracted data from the selected studies. The following key points were collected: first author; publication year; study design; country; total number of cases and subjects; sex; Cd exposure type; and adjusted variables. Adjusted OR was extracted as a preference to non-adjusted OR; however, unadjusted OR and CI were calculated when OR was not provided. When more than one adjusted OR was reported, the ratio with the most number of adjusted variables was selected. Disagreements between authors (JKS and XHY) were resolved through discussion and consensus.

The Newcastle-Ottawa Scale (NOS) was employed to evaluate the methodological quality of each study^42^. The following three primary components were evaluated and assigned a numerical score: (1) study group selection (0–4 points); (2) determination of the exposure source in the study (0–3 points); and (3) adjustment parameters for confounding factors (0–2 points). The total score was nine; a high-quality study was defined as ≥5.

### Statistical analysis

We used OR with 95% CI as the common measure across all studies. Cd caused renal cancer was considered a rare event, the RR and HR in the cohort study was considered approximations of OR. Two articles did not report overall risk estimates, but instead separately presented results for men and women. Therefore, we combined the results using fixed effects and included the pooled risk estimates in the primary analysis^8^,^10^. The OR in two studies failed to extract, so we computed the crude risk estimates and corresponding CI^5^,^7^. The summary risk estimates were calculated using random- or fixed-effects models as appropriate based on heterogeneity levels. Heterogeneity among studies was assessed using the *I*^2^ statistic, which measured quantitative inconsistency in heterogeneity levels across studies. Studies with *I*^*2*^values from 25% to 50% exhibited low heterogeneity, 50% to 75% showed moderate heterogeneity, and studies with results >75% exhibited high heterogeneity. An *I*^2^ value >50% and P_heterogeneity_<0.10 indicated significant heterogeneity. Sensitivity analysis was conducted to evaluate data robustness and stability by sequentially omitting one study on each turn. Studies were sequentially omitted if the data did not meet the restrictions. In addition, subgroup analysis was stratified by study design, effects measure, geographic region, sample size, exposure type, adjustment for variates, and NOS quality.

We evaluated potential publication bias using a funnel plot and Egger’s tests, with *a priori P *< 0.1 indicating a significant publication^43^. If asymmetry evidence was detected, the trim and fill method was employed to correct publication bias^44^. All statistical analyses were conducted using Stata version 13.1 (Stata Corp, College Station, TX, USA).

## Additional Information

**How to cite this article**: Song, J. *et al.* Association between cadmium exposure and renal cancer risk: a meta-analysis of observational studies. *Sci. Rep.* **5**, 17976; doi: 10.1038/srep17976 (2015).

## Figures and Tables

**Table 1:** Characteristic of studies included in the meta-analysis.

Study	Year	Country	Study design	No. of patients	No. of subjects	Sex	Age,Median(Range),yrs	Exposure type	Study period	Adjustment for covariates
Kolonel LN	1976	United States	A case-control study	64	333	M	NA(50-79)	Occupational exposure	1957-1964	Unadjust
Armstrong BG	1985	Britain	A nested case-control study	9	36	W and M	NA(NA)	Occupational exposure	NA	Unadjust
Partanen T	1991	Finland	A case-control study	408	1227	W and M	63(26–95)	Occupational exposure	1977–1978	Unadjust
McCredie M	1993	United States	A population based case-control study	489	1012	W and M	NA(20–79)	Occupational exposure	1989–1991	Adjusted for age, sex, method of interview, and education.
Mandel JS	1995	Mixed countres (Austrial,Denmark,Germany,Sweden and United States)	A multicenter collaborative case-control study	1732	4041	W and M	NA(5–68^+^)	Occupational exposure	1961–1979	Adjusted for age, smoking status, heating oils, kerosene, diesel fuel. body-mass index, education and study center.
PeschB	2000	German and British	A case-control study	935	5233	W and M	NA(40^–^-80^+^)	Occupational exposure	1991–1995	Adjusted for age, study centre, and smoking.
Hu J	2002	Canada	A case-control study	1279	6649	W and M	NA(20–70^+^)	Occupational exposure	1994–1997	Adjusted for 10 year age groups, province, education,BMI(<20, 20-27, >27), pack-years of smoking, alcohol use and total comsuption of meat
Boffetta P	2011	Europe	A hospital-based case-control study	1097	2573	W and M	NA(45^–^–65^+^)	Occupational exposure	1993–2003	Adjusted for gender, age (5-year categories), study centre, and known or suspected risk factors of RCC: place of residence (r ural/urban), tobacco smoking (non-smokers, ex-smokers, and cur rent smokers of 1 e19, 20e 39 and 40 or more pack-years), body mass index (calculated as weight/height 2 and classi fied in five cate-gories: less than 25, 25e 27.4, 27.5 e29.9, 30e 34.9 and 35 or more kg/m 2) and self-reported histor y of hypertension.
Garcia-Esqinas E	2014	United States	A prospective cohort study	25	3792	W and M	56.2 (45–74)	Non-polluted exposure	1989–1991	Adjusted for sex, age, smoking status (never, former, current), cigarette pack-years (continuous), and BMI (<25, 25–30, ≥30 kg/m2).

NA, not available; M, male; W, female.

**Table 2:** Quality assessment of eligible studies based on Newcastle-Ottawa scale.

Author	year	Selection	Comparability	Exposure
Kolonel LN	1976	1	1	2
Armstrong BG	1985	2	1	1
Partanen T	1991	2	1	2
McCredie M	1993	2	1	2
Mandel JS	1995	2	1	3
PeschB	2000	3	2	2
Hu J	2002	3	1	3
Boffetta P	2011	3	1	2
Garcia-Esqinas E	2014	3	2	2

**Table 3:** Results of overall subgroup analysis.

Total	Studies, N	Cases, N	Participants, N	OR (95% CI)	P-value	P of heterogeneity	I^2^ (%)
	10	6038	24896	1.47 (1.27–1.71)	0.000	0.807	0.0
Geographic region							
Europe	5	2513	9402	1.42 (1.20–1.70)	0.000	0.596	0.0
North America	4	1793	11453	1.54 (1.04–2.28)	0.030	0.671	0.0
Mixed population	1	1732	4041	2.00 (1.01–3.95)	0.046	NA	NA
Effect size							
OR	7	3792	116051	1.46 (1.24–1.71)	0.000	0.653	0.0
RR	2	2221	5053	1.63(0.95–2.82)	0.079	0.327	0.0
HR	1	25	3792	1.39 (0.43–4.54)	0.585	NA	NA
Sample size							
Large	7	5940	20735	1.46 (1.24–1.71)	0.000	0.894	0.0
Small	3	98	4161	1.64 (0.83–3.25)	0.155	0.232	31.6
Adjustment for covariates							
Yes	7	5557	23300	1.45 (1.23–1.72)	0.000	0.894	0.0
NO	3	481	1596	1.57 (1.00–2.47)	0.050	0.230	31.9
NOS score							
High	8	5965	24527	1.46 (1.24–1.71)	0.000	0.943	0.0
Low	2	73	369	1.23 (0.21–7.11)	0.817	0.094	64.4
Study design							
Case control study	9	6013	21104	1.47 (1.26–1.72)	0.000	0.726	0.0
Cohort	1	25	3793	1.39 (0.43–4.54)	0.585	NA	NA
Exposure type							
Occupational exposure	9	6013	21104	1.47 (1.26–1.72)	0.000	0.726	0.0
Non-occupational exposurre	1	25	3792	1.39 (0.43–4.54)	0.585	NA	NA
Gender							
Male	3	NA	NA	1.40 (1.16–1.69)	0.001	0.740	0.0
Female	3	NA	NA	1.64 (1.09–2.47)	0.019	0.243	29.3

OR, odds ratio; CI, confidence interval; NA, not available; Large, ≥100 cases; Small, <100 cases; High, NOS score of ≥5; Low, NOS score of <5.
